# Optic atrophy, cataracts, lipodystrophy/lipoatrophy, and peripheral neuropathy caused by a de novo *OPA3* mutation

**DOI:** 10.1101/mcs.a001156

**Published:** 2017-01

**Authors:** Stephanie C. Bourne, Katelin N. Townsend, Casper Shyr, Allison Matthews, Scott A. Lear, Raj Attariwala, Anna Lehman, Wyeth W. Wasserman, Clara van Karnebeek, Graham Sinclair, Hilary Vallance, William T. Gibson

**Affiliations:** 1 Department of Microbiology and Immunology, Dalhousie University, Halifax, Nova Scotia B3H 4R2, Canada;; 2 Department of Medical Genetics, University of British Columbia, Vancouver, British Columbia V6T 1Z4, Canada;; 3 Child and Family Research Institute, British Columbia Children's Hospital, Vancouver, British Columbia V5Z 4H4, Canada;; 4 Faculty of Health Sciences, Simon Fraser University, Vancouver, British Columbia V5A 1S6, Canada;; 5 AIM Medical Imaging, Vancouver, British Columbia V6H 1C9, Canada;; 6 Department of Pathology, British Columbia Children's Hospital, Vancouver, British Columbia V6H 3N1, Canada

**Keywords:** ataxia, congenital nuclear cataract, progressive peripheral neuropathy

## Abstract

We describe a woman who presented with cataracts, optic atrophy, lipodystrophy/lipoatrophy, and peripheral neuropathy. Exome sequencing identified a c.235C > G p.(Leu79Val) variant in the optic atrophy 3 (*OPA3*) gene that was confirmed to be de novo. This report expands the severity of the phenotypic spectrum of autosomal dominant *OPA3* mutations.

## INTRODUCTION

Pathogenic rare variants in *OPA3* have previously been shown to cause optic atrophy, with either autosomal dominant or autosomal recessive inheritance (Sergouniotis et al. 2015). The OPA3 protein primarily localizes at the inner mitochondrial membrane and possibly the outer mitochondrial membrane (Grau et al. 2013). Although the exact function of OPA3 is unknown, previous studies have suggested OPA3 involvement in regulating mitochondrial morphology (Ryu et al. 2010; Grau et al. 2013).

Costeff syndrome, also known as type III 3-methylglutaconic aciduria, is caused by autosomal recessive *OPA3* mutations and, to date, has been reported almost exclusively among patients of Iraqi-Jewish descent (Yahalom et al. 2014). Patients with Costeff syndrome usually present with early-onset bilateral optic atrophy, later-onset spasticity, extrapyramidal dysfunction, cerebellar ataxia, and mild cognitive defect (Anikster et al. 2001; Sofer et al. 2015). A significantly increased level of 3-methylglutaconic acid (>40 µmol/mmol Cr) in the urine of an individual with optic atrophy confirms a diagnosis of Costeff syndrome (Reynier et al. 2004; Pei et al. 2010; Wortmann et al. 2013).

Autosomal dominant *OPA3* mutations causing optic atrophy appear to be less common than the recessive form, and the phenotypes, attributable to these rare variants, are more variable (Sergouniotis et al. 2015). Autosomal dominant optic atrophy (ADOA) caused by an *OPA3* mutation usually presents with optic atrophy and cataracts, although some patients have been seen with isolated optic atrophy (Sergouniotis et al. 2015). Hearing loss, nystagmus, and glaucoma have also been observed in patients with ADOA caused by *OPA3* mutations (Sergouniotis et al. 2015). Peripheral neuropathy and ataxia in these cases were first described in 1961 by Garcin and coworkers among a French family that manifested autosomal dominant inheritance of optic atrophy and cataract. Follow-up studies by Reynier et al. (2004) attributed this family's disorder to a c.277G > A, p.(Gly93Ser) mutation transition in exon 2 of *OPA3*, which cosegregated with the phenotype.

Here we describe a patient with optic atrophy, cataracts, ataxia, and peripheral and autonomic neuropathy associated with an autosomal dominant c.235C > G p.(Leu79Val) mutation in exon 2 of the *OPA3* gene (National Center for Biotechnology Information [NCBI] reference sequence NM_025136.3).

## RESULTS

### Clinical Presentation and Family History

The female Caucasian proband presented with nystagmus at birth and bilateral cataracts that were first detected at 3 mo of age. Her lenses were removed during her first year, but there was no apparent improvement in her best-corrected visual acuity. At age 6 yr, she was found to have optic nerve atrophy after a visual evoked potentials (VEP) test. The VEP test concluded that the patient had highly abnormal visual evoked potentials in both eyes. She was otherwise healthy until 12 yr of age when she started experiencing ataxia, which caused her to stumble. The ataxia initially manifested only in her lower limbs, and then her upper limbs began to show signs of ataxia at ∼15 yr of age. She started experiencing falls at age 17 and was formally diagnosed with peripheral neuropathy after nerve conduction studies were performed at age 21. The nerve conduction studies showed a complete absence of all sensory responses in the upper and lower extremities. Motor nerve conduction studies in the upper limbs were normal, and there were no findings that suggested demyelination.

The patient recalled a relatively rapid progression of her neuropathy between 17 and 27 yr of age, though the progression has slowed for the last few years. Her visual acuity has worsened since birth, decreasing from 20/200 as a young child to 20/450 currently. She also has symptoms of palinopsia with smearing effect, double vision, and visual snow. By age 23 yr, she had cutis marmorata in her extremities, a novel phenotype for *OPA3* mutations, suggesting some degree of vasomotor instability. She also has cerebellar findings including a marked intention tremor, dysdiadochokinesia, and mild dysarthria, suggesting there is a cerebellar component to her ataxia. She has an extrapyramidal component with some chorea, postural tremor, and vocal tremor. She has normal facial features with the exception of upslanting palpebral fissures and is of normal intelligence. The possibility of a secondary lipodystrophy was considered because she also has generalized wasting of muscle and subcutaneous fat, especially in the hands and forearms. She has diminished light touch sensation from knees distally and distal to the metacarpophalangeal joints of the hands. Blood biomarkers have been unremarkable over the last 10 yr, except for occasional mild neutropenia and mild elevations of creatine kinase and total cholesterol (Table 1). Urinalysis has shown moderate ketones and a slightly elevated vitamin E, though urine organic acids found no evidence of 3-methylglutaconic aciduria as her 3-methylglutaconic acid level was 9 mmol/mol of creatinine (reference range <20 mmol/mol Cr). Her electromyography (EMG) showed chronic neurogenic changes, and brain magnetic resonance imagings (MRIs) performed at ages 20 and 23 yr were normal with the exceptions of signs of bilateral optic nerve and chiasmal atrophy. There is no apparent cardiac involvement as demonstrated by a normal electrocardiogram and echocardiogram.

**Table 1.** Serum biomarker concentrations

Biomarker	Patient serum concentration	Reference range
Alanine aminotransferase	22 U/l	<36 U/l
Albumin	46 g/l	35–50 g/l
Alkaline phosphatase	55 U/l	<125 U/l
Aspartate aminotransferase	29 U/l	<36 U/l
Bilirubin	14 µmol/l	3–17 µmol/l
Ceruloplasmin	235 mg/l	220–455 mg/l
Cholesterol	5.0 mmol/l	2.0–4.6 mmol/l
HDL cholesterol	1.6 mmol/l	>1.1 mmol/l
LDL cholesterol	3.1 mmol/l	1.5–3.0 mmol/l
Cholesterol-to-HDL ratio	3.1	>4.4
Copper	13.1 µmol/l	11.3–25.2 µmol/l
Creatine kinase	169 U/l	<140 U/l
Ferritin	15 µg/l	15–200 µg/l
Folate	755 nmol/l	>12 nmol/l
γ-Glutamyl transferase	14 U/l	<31 U/l
Glucose (fasting)	4.7 mmol/l	3.6–6.0 mmol/l
Homocysteine	7.0 mmol/l	<7.8 mmol/l
Lactic acid	0.7 mmol/l	0.5–2.2 mmol/l
Triglyceride	0.6 mmol/l	<2.3 mmol/l
Urea	7 mmol/l	2–9 mmol/l

HDL, high-density lipoprotein; LDL, low-density lipoprotein.

She experiences constant paresthesia and numbness in her extremities. Diminished light touch sensation in her hands and feet make it difficult for her to distinguish textures. She also experiences proximal muscle weakness. She has lost position sense in her distal interphalangeal joints in both her hands and feet. Deep tendon reflexes are absent in her ankles, knees, and wrists. She has hyperesthesia of her lower legs, particularly of the soles of her feet. She also has atrophy of the fat pads of her face and has experienced neuropathic pain in her hands and feet. She has an ataxic gait and has reduced hearing in her left ear (512 mHz). Her parents and two siblings showed no signs of sharing her phenotype.

### Genomic Analysis

**Figure 1.**
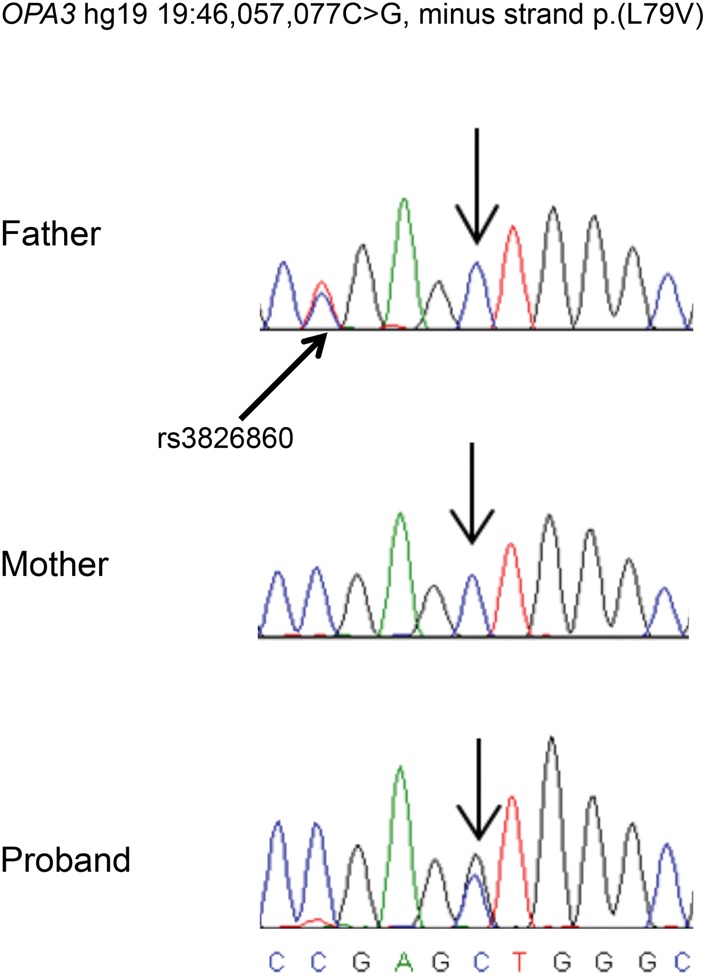
Sanger sequencing traces with arrows indicating the mutation and a nearby single-nucleotide polymorphism (SNP). Traces show that the *OPA3* variant (Chr19:46,057,077C > G p.(L79V) annotated per hg19) is de novo in the proband. The father is heterozygous for rs3826860, a synonymous variant that is annotated in the Database for Short Genetic Variations (dbSNP) at a nearby nucleotide (g.46,057,081A > G; c.231T > C; p.(Ala77=)); the proband and her mother are homozygous for the SNP, whereas only the proband is heterozygous for the mutation.

At age 20, medical genetics first assessed her to search for a potential diagnosis. She had normal chromosomes and a normal test for Friedreich's ataxia. Genes associated with Charcot–Marie–Tooth disease (*MPZ*, *MFN2*, and *GJB1*) were sequenced also, as Charcot–Marie–Tooth disease presents with progressive atrophy and muscle weakness secondary to neuropathy (Roa et al. 1996; Züchner et al. 2004; Rouzier et al. 2012). The common duplication of *PMP22* was ruled out by clinical-grade testing, and genes known to cause lipodystrophy (*LMNA*, *PPARG*, *EMD*, *AGPAT2*, *BSCL2*) were also sequenced. No pathogenic variants were found in any of these genes. Testing of *OPA3* as a candidate gene by an external laboratory on a research basis did not identify any rare variants, so trio-based exome sequencing was performed on blood-derived DNA from the patient and her parents. The sequencing results identified a de novo missense variant (c.235C > G p.[Leu79Val]) in the patient's *OPA3* gene (NCBI reference sequence NM_025136.3), a gene known to cause optic atrophy and cataracts (Reynier et al. 2004; Ayrignac et al. 2012). Sanger confirmation of the variant of interest was carried out using standard polymerase chain reaction (PCR) in the proband and in parental DNA. Sanger sequencing confirmed that the proband was heterozygous for the variant and that the variant was absent from both parents (Fig. 1). The variant is absent from the Database for Short Genetic Variations (dbSNP) build 146, National Heart, Lung, and Blood Institute (NHLBI) Exome Variant Server, and Exome Aggregation Consortium (ExAC) (Table 2). The genomic position is highly conserved in vertebrates and is predicted to be damaging by Combined Annotation-Dependent Depletion (CADD) (CADD version 1.3 score was 19.02), MutationTaster (probability 0.999311228255692), and Protein Variation Effect Analyzer (PROVEAN) software (PROVEAN score was −3.00, with anything less than −2.5 considered to be deleterious; Choi 2012; Choi et al. 2012). *OPA3* has a residual variation intolerance score (RVIS) of 20.96, which suggests it is not a gene that is frequently seen to harbor rare benign mutations, thereby further supporting the pathogenicity of our de novo variant. Parental relatedness to the proband was confirmed. There were no other variants detected in any genes that are known to cause Charcot–Marie–Tooth disease or other diseases with a similar phenotype (average read coverage, 37.5×).

**Table 2.** *OPA3* variant summary

Gene	Chr. (hg19)	Position	Mutation	Amino acid change	Inheritance	Public database reference^a^
*OPA3*	19	g.46,057,077G > C	NM_025136.3	p.Leu79Val	De novo	Not described

^a^Public databases checked include Database for Short Genetic Variations (dbSNP) build 146, National Heart, Lung, and Blood Institute (NHLBI) Exome Variant Server, and Exome Aggregation Consortium (ExAC).

## DISCUSSION

The patient presented with an unusually severe phenotype for autosomal dominant optic atrophy caused by an *OPA3* mutation. Certain aspects of her phenotype have been observed in others with heterozygous mutations in this gene, though rarely to this degree. In addition to the known features of optic atrophy and cataracts, she has peripheral motor and sensory neuropathy as well as evidence of autonomic neuropathy. Although neurological symptoms, such as extrapyramidal signs and ataxia, have been associated with autosomal dominant *OPA3* mutations, this is the first documented case of severe neuropathy (Garcin et al. 1961; Reynier et al. 2004). She also has occasional difficulties swallowing and frequent episodes of abdominal pain with alternating constipation and diarrhea. Gastrointestinal symptoms were also reported in a French family with a heterozygous c.313C > G, p.(Gln105Glu) *OPA3* mutation (Ayrignac et al. 2012).

The patient also has periodic lightheadedness, urinary symptoms, and a variable body temperature further suggesting autonomic nervous system involvement. Her urine organic acid profile (run twice on two different occasions) was normal with the exception of moderate ketone levels and the presence of myoglobin. Normal organic acid profiles are not uncommon in individuals with autosomal dominant *OPA3* mutations (Reynier et al. 2004). She has pain in her extremities, reduced hearing in her left ear, and gait abnormalities, which are all previously associated with mutations in *OPA3* (Ayrignac et al. 2012; Sergouniotis et al. 2015). She has also experienced rapid weight loss since age 20 and is now in the <5th percentile of weight for her height. Metabolic testing was performed and the results showed that her resting energy expenditure was ∼84% of that predicted for her age, but an adjusted resting energy expenditure was calculated and was relatively high compared to controls similar to her size (Supplemental Tables S1–S4). Whether she has true lipodystrophy (abnormal growth and/or loss of adipose tissue due to a primary, intrinsic defect of the tissue) as opposed to lipoatrophy (loss of adipose tissue secondary to hypermetabolism) could not be established with certainty, though her relatively high metabolic rate suggests the possibility of the former. Mouse models with a homozygous mutation in *Opa3* showed a 60% reduction in body weight and profound intra-abdominal leanness, suggesting that the patient's loss of adipose tissue may be caused by her *OPA3* mutation (Davies et al. 2008; Wells et al. 2012; Navein et al. 2016). The atypical set of phenotypes observed in this case expands the phenotypic spectrum of autosomal dominant mutations in *OPA3* and identifies a de novo mutation, which we believe to be pathogenic for these phenotypes. However, additional functional studies or cases with a similar phenotype will be necessary to confirm the pathogenicity of autosomal dominant variants in *OPA3*.

## METHODS

### Exome Sequencing

Genomic DNA was extracted from whole blood collected from the affected patient and her parents and trio-based exome sequencing was performed at PerkinElmer corporation using Agilent V4 51Mb capture with sequencing of 100-bp paired-end reads on an Illumina HiSeq 2000 at average 30× coverage across the known exons (Table 3). Data analysis was carried out using an in-house bioinformatics pipeline to align the FASTQ reads to the human genome reference version 19 (GRCh37.75) and to identify and assess the predicted effect of rare (<3% allelic frequency from dbSNPv146) variants on protein function (Shyr et al. 2014). De novo dominant-, recessive-, and X-linked modes of inheritance were assessed through in-house scripts, and variants altering the protein code were selected (missense, nonsense, frameshift changes, in-frame deletions, and splice-site effects) (Table 4). Remaining variants were quality-screened manually using the Integrative Genomics Viewer (https://www.broadinstitute.org/igv/). The variants were narrowed down based on their clinical significance and similarity to the phenotype.

**Table 3.** Sequence coverage

Sample	Number of starting paired-end reads	Percentage of reads aligned	Median read coverage	Percentage of coding sites with >20-fold coverage	Percentage of *OPA3* sites with >10-fold coverage
Proband	67,382,674	82.1	37.5	93.5	100
Mother	61,340,881	79.3	33.6	90.1	100
Father	59,968,033	83.6	32.1	88.6	100

**Table 4.** Variants identified from exome sequencing

	Filtering results	Manual review	Resulting genes of interest
Homozygous (# seq. changes)	4 (4)	0 (0)	0 (0)
Compound heterozygous (# seq. changes)	8 (16)	0 (0)	0 (0)
De novo genes (# seq. changes)	28 (28)	2 (2)	1 (1)
X-linked genes (# seq. changes)	9 (9)	1 (1)	0 (0)
Total genes (# seq. changes)	49 (57)	3 (3)	1 (1)

### Sanger Sequencing

Sanger sequencing of the variant of interest within the *OPA3* gene was carried out on DNA amplified by standard PCR. PCR reactions were prepared in a 20 µl volume with 40 ng genomic DNA, 1× GoTaq Green Mastermix (Promega), 5% dimethyl sulfoxide (DMSO) (Sigma-Aldrich), and 0.25 µM of the primer pair. Primers were designed by Life Technologies (*OPA3*, Hs00760967_CE; forward, 5′-GCCAGTACTCCAGCACTAGG-3′, reverse, 5′-CTGCATTCCCTGGGTGAGAG-3′). The PCR began with a 2-min cycle at 94°C, followed by 35 cycles of 45 sec at 94°C, 45 sec at 63°C, and 1 min at 72°C, ending with extension for 10 min at 72°C. Sanger sequencing was carried out on an ABI 3130xl.

### Indirect Calorimetry

The canopy (dilution) method was used to obtain indirect calorimetry information. Ambient and diluted fractions of O_2_ and CO_2_ were measured for a known ventilation rate. O_2_ consumption and CO_2_ production were determined and converted into resting energy expenditure. The abbreviated Weir equation (without urinary nitrogen) was used to convert gas exchange measures into resting energy expenditure.

## ADDITIONAL INFORMATION

### Data Deposition and Access

Exome sequencing data is not publicly available because patient consent could not be obtained. The variant found in the proband has been deposited into ClinVar (http://www.ncbi.nlm.nih.gov/clinvar/) under accession number SCV000266846.

### Ethics Statement

Written informed consent was obtained for collecting blood samples and sequencing from all study participants. The studies conducted here were approved by the Institutional Review Boards of Simon Fraser University and of UBC/Children's and Women's Health Centre of British Columbia.

### Acknowledgments

We thank the patient and her family for their generous contribution. We also gratefully acknowledge Selina Gyawali (Simon Fraser University) and Wayne Picker (AIM Medical Imaging) for performing the imaging studies.

### Author Contributions

S.C.B. analyzed the data, drafted, and critically reviewed the manuscript. K.N.T. analyzed the data and critically reviewed the manuscript. C.S., A.L., and A.M. provided and analyzed the exome data. R.A. and S.A.L. analyzed the imaging studies. W.W.W. and C.v.K. analyzed the data. G.S and H.V. analyzed the urine organic acids data. W.T.G. conceived of the study, performed multiple clinical assessments, analyzed the data, and drafted and critically reviewed the manuscript.

### Funding

This work was supported by the Canadian Institutes of Health Research (CIHR; Operating Grants PCN102990, PCN110794, MOP119595) and by Clinician Scientist salary awards to W.T.G. (CIHR and Child and Family Research Institute).

### Competing Interest Statement

The authors have declared no competing interest.

## Supplementary Material

Supplemental Material
