# Supplementary material for: Optic atrophy, cataracts, lipodystrophy/lipoatrophy, and peripheral neuropathy caused by a de novo OPA3 mutation
Source: Cold Spring Harb Mol Case Stud. 2017 Jan;3(1):a001156. doi: 10.1101/mcs.a001156 (PMC5171695; doi:10.1101/mcs.a001156)
Supplement: Supplemental Material [file supp_3_1_a001156__index.html]

Optic atrophy, cataracts, lipodystrophy/lipoatrophy, and peripheral neuropathy caused by a de novo OPA3 mutation — Supplemental Material 

# Optic atrophy, cataracts, lipodystrophy/lipoatrophy, and peripheral neuropathy caused by a de novo *OPA3* mutation

## Supplemental Material

**Files in this Data Supplement:**

- Supplemental Tables 1-4.docx
