# Supplementary material for: Optic atrophy, cataracts, lipodystrophy/lipoatrophy, and peripheral neuropathy caused by a de novo OPA3 mutation
Source: Cold Spring Harb Mol Case Stud. 2017 Jan;3(1):a001156. doi: 10.1101/mcs.a001156 (PMC5171695; doi:10.1101/mcs.a001156)
Supplement: Supplemental Material [file supp_mcs.a001156_Supplemental_Tables_1-4.docx]

Supplementary Table S1. Body composition results (raw data).

_________________________________________________________________________________________________

Anatomical Bone mineral Fat Lean Fat-free Total mass (g) Percent Percent

region content (g) mass (g) mass (g) mass (g) fat fat^a^

(control)

_________________________________________________________________________________________________

Left arm 118.14 234.1 1346.8 1464.9 1699.4 13.8 34.2

Right arm 137.42 277.6 1394.8 1532.2 1809.8 15.3 29.1

Trunk 512.26 3106.6 16304.8 16817.1 19923.6 15.6 26.3

Left leg 523.93 2977.1 3059.2 3583.1 6560.1 45.4 35.3

Right leg 293.43 3001.9 2802.4 3095.8 6097.7 49.2 34.1

Subtotal 1585.18 9597.6 24907.9 26493.1 36090.7 27.9 31.8

Head 717.99 736.7 2832.2 3550.2 4286.9 17.2 18.3

Total 2303.17 10334.3 27740.2 30043.3 40377.6 26.1 29.6

_________________________________________________________________________________________________

*The patient is right-hand dominant.

*Note: All values were measured using Dual-energy X-ray absorptiometry (DXA).

^a^From 7 healthy females aged 21-28.

Supplementary Table S2. Bone mineral density.

________________________________________________________________________________

Anatomical region Area Bone mineral Bone mineral Control bone

(cm^3^) content (g) density (g/cm^2^) mineral density^a^

(g/cm^2^)

________________________________________________________________________________

Left arm 157.64 118.14 0.749 0.714

Right arm 181.83 137.42 0.756 0.680

Left ribcage 115.11 77.49 0.673 0.673

Right ribcage 115.89 75.30 0.650 0.605

Thoracic spine 116.28 101.00 0.869 0.834

Lumbar spine 43.31 46.51 1.074 1.034

Pelvis 218.90 211.96 0.968 1.015

Left leg 305.92 523.93 1.713 1.132

Right leg 299.68 293.43 0.979 1.111

Head 234.90 717.99 3.057 2.315

_________________________________________________________________________________

*Note: All values were measured using Dual-energy X-ray absorptiometry (DXA).

^a^From 7 healthy females aged 21-28.

Supplementary Table S3. Indirect calorimetry results.

__________________________________________________________________________________________

Respiratory Predicted Measured Resting Lean tissue Adjusted resting Control resting

quotient^a^ metabolic resting energy mass (kg) expenditure^d^  expenditure^e^

rate^b^ expenditure^c^ expenditure (kcal/kg lean (Kcal/kg/day)

(Kcal/day) (Kcal/day) (% of predicted) mass/day)

__________________________________________________________________________________________

0.80 1215 1022 84 27.74 36.9 27.8

__________________________________________________________________________________________

^a^Measured after an overnight fast.

^b^Calculated using the Harris-Benedict equation:

BMR = 655.1 + (9.563 x weight in kg) + (1.850 x height in cm) – (4.676 x age in years)

^c^Calculated using the Abbreviated Weir equation (without urinary nitrogen):

REE (kcal/day) = [3.94(VO2) + 1.1(VCO2)] x 1440, (VO2 = 0.147, VCO2 = 0.117).

^d^Adjusted resting expenditure was calculated by dividing the measured resting expenditure by the measured lean tissue mass.

^e^Calculated using the equation for estimating resting energy expenditure in adult women:

9.99 x (weight in kg) + 6.25 x (height in cm) – 4.94 x (age in years) -161, this value was then divided by the patient’s

weight in kg.

Supplementary Table S4. MRI lipodystrophy results.

_________________________________________________________________________________________________

Anatomical Result Anatomical Result

region region

_________________________________________________________________________________________________

Scalp Thin Breast tissue Entirely glandular with thin layer

Of subcutaneous fat

Temporal Thin at 1.2 cm

Pancreas Unremarkable

Extra-ocular depots Thin fat external to

Extra-ocular muscles Retroperitoneal fat Decreased

Temporalis thickness 3 mm Mesenteric Decreased

Masseter thickness 3 mm Large muscle Groups No fat infiltration

Submental depot Subjectively negligible fat Pericardial region Subjectively normal

Dorsal to C7 Thin at 5 mm Biceps femoris, Infiltrated with fat

Plantaris, and soleus

Anterior to Thin at 4 mm

acromioclavicular joint

Anterior to Negligible Upper extremities Subcutaneous tissue

sternomanubrial joint appears normal

Around great vessels Negligible Lower extremities Subcutaneous tissue

appears normal

_________________________________________________________________________________________________________________________________________________
